# Characterization and phylogenetic analysis of the complete mitochondrial genome sequence of *Lagenaria siceraria*, a cucurbit crop

**DOI:** 10.3389/fpls.2025.1599596

**Published:** 2025-07-22

**Authors:** Xuan Du, Kuanhong Wang, Yuying Tang, Jue Wu, Xiaofeng Yang, Hongmei Zhang, Na Liu, Zhaohui Zhang

**Affiliations:** ^1^ Shanghai Academy of Agricultural Sciences, Shanghai Key Laboratory of Protected Horticultural Technology, Shanghai, China; ^2^ Fengxian District Agricultural Technology Extension Center, Shanghai, China

**Keywords:** *Lagenaria siceraria*, mitochondrial genome, phylogenetic analysis, cucurbit, evolutionary analysis

## Abstract

Bottle gourd (*Lagenaria siceraria*)belongs to cucurbit crop and hasunique semi-autonomous organelle genome. Using Illumina short-read and Nanopore long-read sequencing data, we sequenced and annotated the complete mitochondrial genome of *L. siceraria*. And a comparative phylogenetic analysis was conducted with its close relatives. The mitochondrial genome of bottle gourd is a circular sequence of 357,496 bp with a GC content of 45.03%. It contains 63 genes, including 34 mRNAs, 24 tRNAs, 4 rRNAs, and 1 pseudogene. The *rps19* gene is present, but *rpl10* is absent. 22,294 bp (6.24%) are repetitive sequences. 497 RNA editing sites were identified. 45 homologous fragments (40,579 bp, 11.35%) were shared with the chloroplast genome. Phylogenetic analysis revealed that *C. maxima*, *C. sativus*, *C. lanatus*, and *L. acutangula* are closely related to bottle gourd. Gene arrangement analysis indicated that *L. acutangula* exhibits the highest collinearity with *L. siceraria* compared to other cucurbit crops. However, genome size and repetitive sequences are most similar to watermelon. Nearly all Ka/Ks ratios <1.0 suggest stabilizing selection in protein-coding genes. These findings provide a foundation for further understanding the evolutionary relationships within cucurbit crops.

## Introduction

Plant mitochondria, like chloroplasts, are crucial organelles in plant cell activities, with genomes that are independent of nuclear genomes, exhibiting semi-autonomous genetic characteristics (Rodríguez-Moreno et al., 2011; Wang et al., 2025). Mitochondria play a vital role in plant growth and development plants (Srivastava et al., 2018; Wang et al., 2024a), primarily through their involvement in energy metabolism, providing ATP for cell growth, division, differentiation, metabolism, and apoptosis via oxidative phosphorylation (Møller et al., 2021; Xu et al., 2022). During evolution, plant mitochondrial (mt) genomes have undergone significant changes in gene sequence, genome structure, and sequence migration from other organelles (Greiner and Bock, 2013; Timmis et al., 2004; Chevigny et al., 2020; Kubo and Newton, 2008; Wang et al., 2024a). Consequently, plant mt genomes are 100 to 10,000 times larger than those of animals and exhibit greater structural complexity (Best et al., 2020; Christensen, 2013; Wu et al., 2025). Mitochondrial genomes vary not only among plant species, but also within the same species (O’Conner and Li, 2020; Kozik et al., 2019), in contrast to the highly conserved structure of plant chloroplast genomes (Niu et al., 2023). As a result, mt genomes have become a valuable source of genetic information and have been widely used in phylogenetic studies to understand basic cellular processes (Cao et al., 2023; Xu et al., 2013; Wang et al., 2024b).

Bottle gourd (*Lagenaria siceraria*) (2n = 2x =22), also known as long calabash, belongs to the *Cucurbitaceae* family, which comprises 95 genera and 942–978 species (Tanaka et al., 2013), including cucumber (*Cucumis sativus*), melon (*Cucumis melo*), watermelon (*Citrullus lanatus*), pumpkin (*Cucurbita moschata*) and zucchini (*Cucurbita pepo*). The economic importance of cucurbit crops is second only to that of the *Solanaceae* family (Rodríguez-Moreno et al., 2011).Cucurbit crops are known to possess unique semi-autonomous organelle genomes (mitochondria and chloroplast genomes), with significant differences observed among different species (Levi et al., 2006; Rodríguez-Moreno et al., 2011). Organelle genes in cucurbit crops are associated with critical metabolic pathways such as photosynthesis and respiration, as well as important traits like cold resistance (Olechowska et al., 2022)and sex differentiation (Levi et al., 2006). Mitochondrial genome data can enhance cucurbit breeding programs by identifying conserved genes linked to stress tolerance or yield. Additionally, comparative analyses aid biodiversity conservation by clarifying genetic relationships among species and detecting adaptive traits in wild relatives.

With the advancement of long-read sequencing technologies, organelle genome sequencing has become more accessible. In this study, we constructed and annotated the complete mitochondrial genome of bottle gourd using a combination second- and third-generation sequencing technologies, performed phylogenetic analyses, and compared the mitochondrial genomes of bottle gourd with other cucurbit crops. These results provide insights into the characteristics of the bottle gourd mitochondrial genome and offer a theoretical foundation for further studies on organelle genome differences, evolutionary relationships, and mitochondrial genetic patterns among cucurbit crops.

## Materials and methods

### Plant materials and DNA sequencing

The bottle gourd variety “BG-54” used in this study was obtained commercially from Zhongziku APP (http://www.zhongziku.cc/). The plants were cultivated under controlled conditions at the Zhuanghang Comprehensive Experimental Station(E 121°28′, N 30°57′) of the Shanghai Academy of Agricultural Sciences. The photon flux density ranged from 650 to 850 W.m^-2^ with temperatures between 10–25°C and relative humidity of 50–70%. Fresh leaves were frozen in liquid nitrogen and stored at -80°C. Total DNA was isolated following the protocol for the Illumina NovaSeq 6000 platform (Illumina, San Diego, CA, USA) and the Oxford Nanopore PromethION (Oxford Nanopore Technologies, Oxford Science Park, UK).

**Figure 1:**
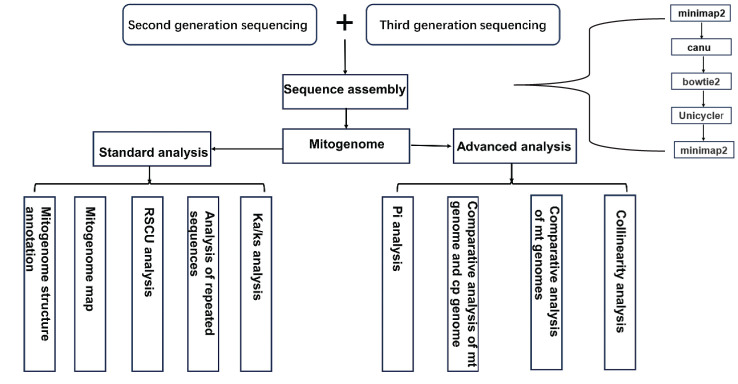
A flow diagram of experiment and analysis.

Raw data from second-generation sequencing were filtered using fastp software (version 0.20.0, https://github.com/OpenGene/Fastp) (Chen et al., 2018). Third-generation sequencing data of mitochondrial reads were filtered using Filtlong (version 0.2.1, https://github.com/rrwick/Filtlong). The filtered third-generation data were aligned to the reference gene sequence using Minimap2 (version 2.1) (Li and Birol, 2018), and sequences with alignment lengths greater than 50 bp were selected. Sequences with overlaps greater than 1 kb and similarity greater than 70% were chosen as seed sequences. The original data were iteratively compared to the seed sequences to obtain all third-generation sequencing data of the mitochondrial genome. The third-generation assembly software Canu (Koren et al., 2017) was used to correct the obtained data. The corrected sequences were then aligned with the second-generation data using Bowtie2 (v2.3.5.1), and Unicycler (v0.4.8) was used to assemble the second- and third-generation data. Due to the complex physical structure of the mitochondrial genome, including subrings and non-circular forms, the corrected third-generation sequencing data were manually compared with the contigs obtained in the second step using Minimap2 to determine the branching direction and obtain the final assembly result (**Figure 1**).

### Mitogenome annotation

Protein-coding genes and rRNA sequences were annotated by comparing them with published plant mitochondrial sequences using BLAST, followed by manual adjustments based on related species. Transfer RNA (tRNA) genes were annotated using tRNAscan-SE (http://lowelab.ucsc.edu/tRNAscan-SE/) (Chan and Lowe, 2019). Open Reading Frames (ORFs) were identified using the Open Reading Frame Finder (http://www.ncbi.nlm.nih.gov/gorf/gorf.html), with the minimum length set to 102 bp. Redundant sequences and those overlapping with known genes were excluded. Sequences with alignments longer than 300 bp were annotated against the nr library. Potential RNA editing sites in the protein-coding genes (PCGs) of bottle gourd were predicted using the online Predictive RNA Editor for Plant Mitochondrial Genes (PREP-Mt) suite (http://prep.unl.edu/) (Mower, 2005). The physical circular map of the mitochondrial genome was generated using the Organellar Genome DRAW (OGDraw) v1.2 program (https://chlorobox.mpimp-golm.mpg.de/OGDraw.html). Relative synonymous codon usage (RSCU) was calculated using the CAI Python package developed by Lee (Lee, 2018),and codon frequencies were determined using the Codon Usage tool in the Sequence Manipulation Suite (bioinformatics.org/sms2/codon_usage.html) (Stothard, 2000).

### Analysis of repeated sequences

Three types of repeats—simple sequence repeats (SSRs), tandem repeats, and dispersed repeats—were identified in the bottle gourd mitochondrial genome. SSRs were detected using the MIcroSAtellite identification tool (v1.0, parameters: 1-10 2-5 3-4 4-3 5-3 6-3) implemented in a Perl script (Thiel et al., 2003). Tandem repeats (>6 bp repeat units) were identified using Tandem Repeats Finder v4.09 (trf409.linux64, parameters: 27 7 80 10 50 2000 -f -d -m) (http://tandem.bu.edu/trf/trf.submit.options.html) (Benson, 1999). Dispersed repeats were detected using BLASTn (v2.10.1) with the following parameters: -word_size 7 and E-value 1e-5.

### Ka/Ks analysis

Gene sequences were aligned using MAFFT V7.310 (https://mafft.cbrc.jp/alignment/software/), and the nonsynonymous-to-synonymous substitution ratio (Ka/Ks) was calculated using the Ka/Ks Calculator V2.0 (https://sourceforge.net/projects/kakscalculator2/). The MLWL method was employed for the calculations.

### Pi analysis

Nucleotide diversity (Pi) was used to assess sequence variation among different species, with regions of high variation serving as potential molecular markers for population genetics. Homologous gene sequences from different species were globally aligned using MAFFT software (v7.427, –auto mode). The aligned sequences were concatenated, trimmed using trimAl (v1.4.rev15, parameter: -gt 0.7), and analyzed with DNAsp5 to calculate Pi values for each gene.

### Homologous sequence analysis of chloroplast and mitochondria

Homologous sequence analysis between chloroplast and mitochondrial genomes were conducted using BLAST, with a similarity threshold of 70% and an E-value of 1e-5. The results were visualized using Circos v0.69-5.

### Phylogenetic tree construction and sequence collinearity analysis

Phylogenetic analysis was conducted using the mitochondrial genomes of bottle gourd and 32 other species representing 24 families. Sequences from different species were aligned using MAFFT software (v7.427, –auto mode). The aligned sequences were concatenated, trimmed with trimAl (v1.4.rev15, parameter: -gt 0.7), and the best-fit evolutionary model (GTR) was determined using jModelTest-2.1.10. A maximum likelihood phylogenetic tree was constructed using RAxML V8.2.10 (https://cme.h-its.org/exelixis/software.html) under the GTRGAMMA model with 1,000 bootstrap replicates.

Collinearity analysis of the bottle gourd mitochondrial genome was performed using two methods. The first method involved comparing genomes using nucmer (4.0.0beta2) with the –maxmatch parameter to generate dot-plot diagrams. The second method utilized BLASTn (v2.10.1+) with parameters set to -word_size 7 and E-value 1e-5. Fragments with alignment lengths greater than 300 bp were screened, and collinearity maps were generated by comparing the assembled species with selected species.

## Results

### Features of the bottle gourd mitogenome

**Figure 2:**
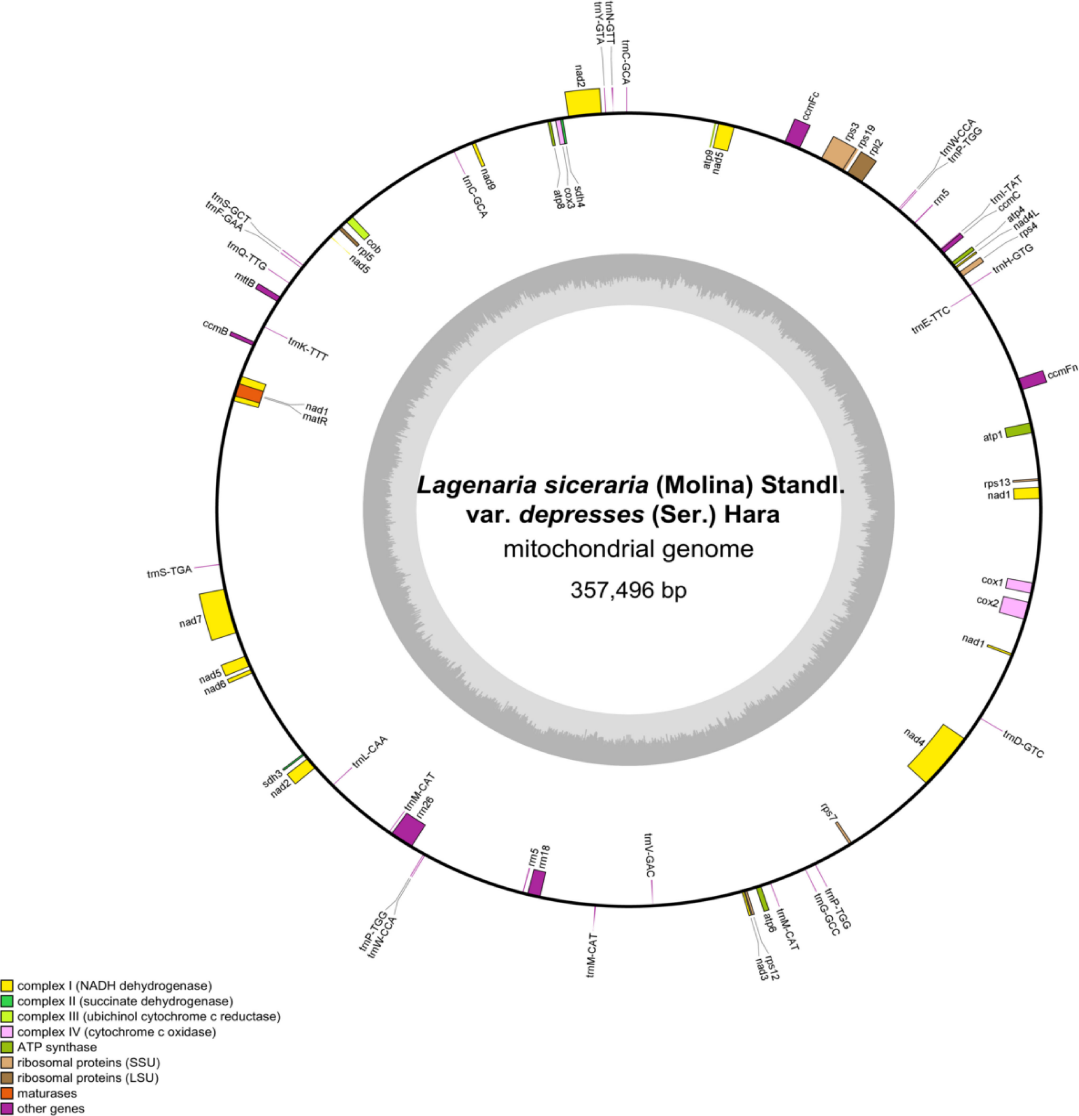
*L. siceraria* mitogenome gene map. Genes shown on the outside and inside of the circle are transcribed clockwise and counterclockwise, respectively. The dark gray region in the inner circle depicts GC content.

The Illumina MiSeq and Nanopore sequencing produced 29,675,595 and 1,406,000 reads, respectively, with a mean read length of 7,433 bp for Nanopore sequencing. The complete mitochondrial genome of bottle gourd is a circular sequence of 357,496 bp with a GC content of 45.03% (**Figure 2**). The sequence has been submitted to the GenBank database (accession number: PP727017). The mitochondrial genome contains 63 genes, including 34 mRNAs, 24 tRNAs, 4 rRNAs, and 1 pseudogene (**Table 1**). Notably, three copies of the *nad1* and *nad5* genes were identified. Additionally, three tRNA genes located in repeat regions were found in two or three copies (*trnp-TGG*, *trnM-CAT*, and *trnW-CCA*) (**Figure 2**).

**Table 1:** Gene profile and organization of the bottle gourd mitogenome.

Group of genes	Gene name	Length	Start codon	Stop codon	Amino acid
ATP synthase	atp1	1530	ATG	TGA	510
	atp4	597	ATG	TAG	199
	atp6	765	ATG	TAA	255
	atp8	480	ATG	TAA	160
	atp9	225	ATG	CGA(TGA)	75
Cytohrome c biogenesis	ccmB	621	ATG	TGA	207
	ccmC	699	ATG	TGA	233
	ccmFc	1317	ATG	TGA	439
	ccmFn	1734	ATG	TGA	578
Ubichinol cytochrome c reductase	cob	1173	ATG	TAG	391
Cytochrome c oxidase	cox1	1584	ACG(ATG)	TAA	528
	cox2	783	ATG	TAA	261
	cox3	798	ATG	TGA	266
Maturases	matR	1947	ATG	TAG	649
Transport membrance protein	mttB	849	ATG	TAG	283
NADH dehydrogenase	nad1	978	ACG(ATG)	TAA	326
	nad2	1467	ATG	TAA	489
	nad3	357	ATG	TAA	119
	nad4	1488	ATG	TGA	496
	nad4L	303	ACG(ATG)	TAA	101
	nad5	2001	ATG	TAA	667
	nad6	618	ATG	TAA	206
	nad7	1185	ATG	TAG	395
	nad9	573	ATG	TAA	191
Ribosomal proteins (LSU)	rpl2	1008	ATG	TAA	336
	rpl5	558	ATG	TAA	186
Ribosomal proteins (SSU)	rps12	378	ATG	TGA	126
	rps13	351	ATG	TGA	117
	rps19	279	ATG	TAA	93
	rps3	1692	ATG	TAG	564
	rps4	831	ATG	TAA	277
	rps7	447	ATG	TAA	149
Succinate dehydrogenase	sdh3	309	ATG	TAA	103
	sdh4	387	ATG	CGA(TGA)	129
Ribosomal RNAs	rrn18	1857	_	_	_
	rrn26	3375	_	_	_
	rrn5	112	_	_	_
	rrn5	121	_	_	_
Transfer RNAs	trnC-GCA	71	_	_	_
	trnC-GCA	73	_	_	_
	trnD-GTC	74	_	_	_
	trnE-TTC	72	_	_	_
	trnF-GAA	74	_	_	_
	trnG-GCC	72	_	_	_
	trnH-GTG	74	_	_	_
	trnI-TAT	76	_	_	_
	trnK-TTT	73	_	_	_
	trnL-CAA	81	_	_	_
	trnM-CAT	74	_	_	_
	trnM-CAT	77	_	_	_
	trnM-CAT	73	_	_	_
	trnN-GTT	72	_	_	_
	trnP-TGG	74	_	_	_
	trnP-TGG	75	_	_	_
	trnP-TGG	75	_	_	_
	trnQ-TTG	72	_	_	_
	trnS-GCT	88	_	_	_
	trnS-TGA	87	_	_	_
	trnV-GAC	72	_	_	_
	trnW-CCA	74	_	_	_
	trnW-CCA	74	_	_	_
	trnY-GTA	83	_	_	_

### Codon usage analysis of PCGs

In the mitochondrial (mt) genome of bottle gourd, the protein-coding genes (PCGs) can be categorized into 10 functional groups. These include ATP synthases (5 genes), cytochrome C biogenesis accessory proteins (4 genes), ubiquinol cytochrome C reductases (1 gene), cytochrome C oxidases (3 genes), maturases (1 gene), transport membrane proteins (1 gene), NADH dehydrogenases (9 genes), ribosomal proteins (LSU) (2 genes), ribosomal proteins (SSU) (6 genes), and succinate dehydrogenases (2 genes). Most PCGs utilize the typical ATG start codon, while *cox1*, *nad1*, and *nad4L* begin with ACG, likely due to C-to-U RNA editing at the second codon position (**Table 1**). Four types of stop codons were identified: TGA, TAG, TAA, and CGA. RNA editing from C to U was observed in the stop codons of *atp9* and *sdh4* (**Table 1**). The usage frequencies of these stop codons were 26.47% (TGA), 17.65% (TAG), 50% (TAA), and 5.88% (CGA), with TAA being the most frequently used stop codon.

**Figure 3:**
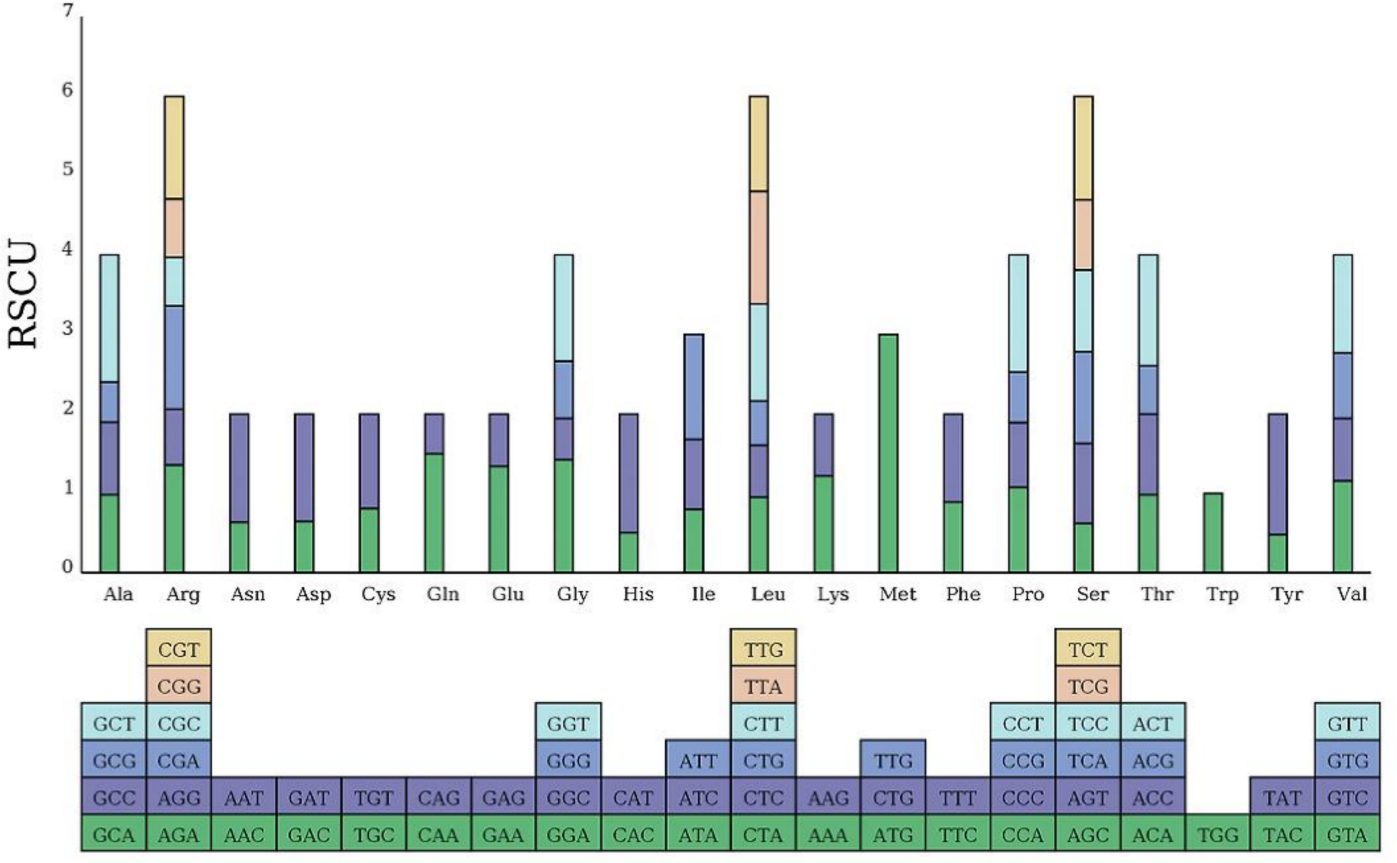
Relative synonymous codon usage (RSCU) in the *L. siceraria* mitogenome. Codon families are shown on the x-axis. RSCU values are the number of times a particular codon is observed relative to the number of times that codon would be expected for a uniform synonymous codon usage.

The coding sequence (CDS) length of the bottle gourd mitochondrial genome is 30,212 bp, encoding 10,104 codons. Among these, 31 codons exhibited a relative synonymous codon usage (RSCU) value greater than 1, indicating a higher usage frequency compared to other synonymous codons. Analysis of RSCU for 24 PCGs in the bottle gourd mitogenome revealed that all NNT and NNA codons had RSCU values exceeding 1.0, except for the termination codon TGA (0.97) and the alanine codon GCA (0.98) (**Figure 3**). Codon usage in the bottle gourd mitogenome showed a strong bias toward A or T(U) at the third codon position, a pattern commonly observed in the mitochondrial genomes of land plants.

### Prediction of RNA editing sites

**Figure 4:**
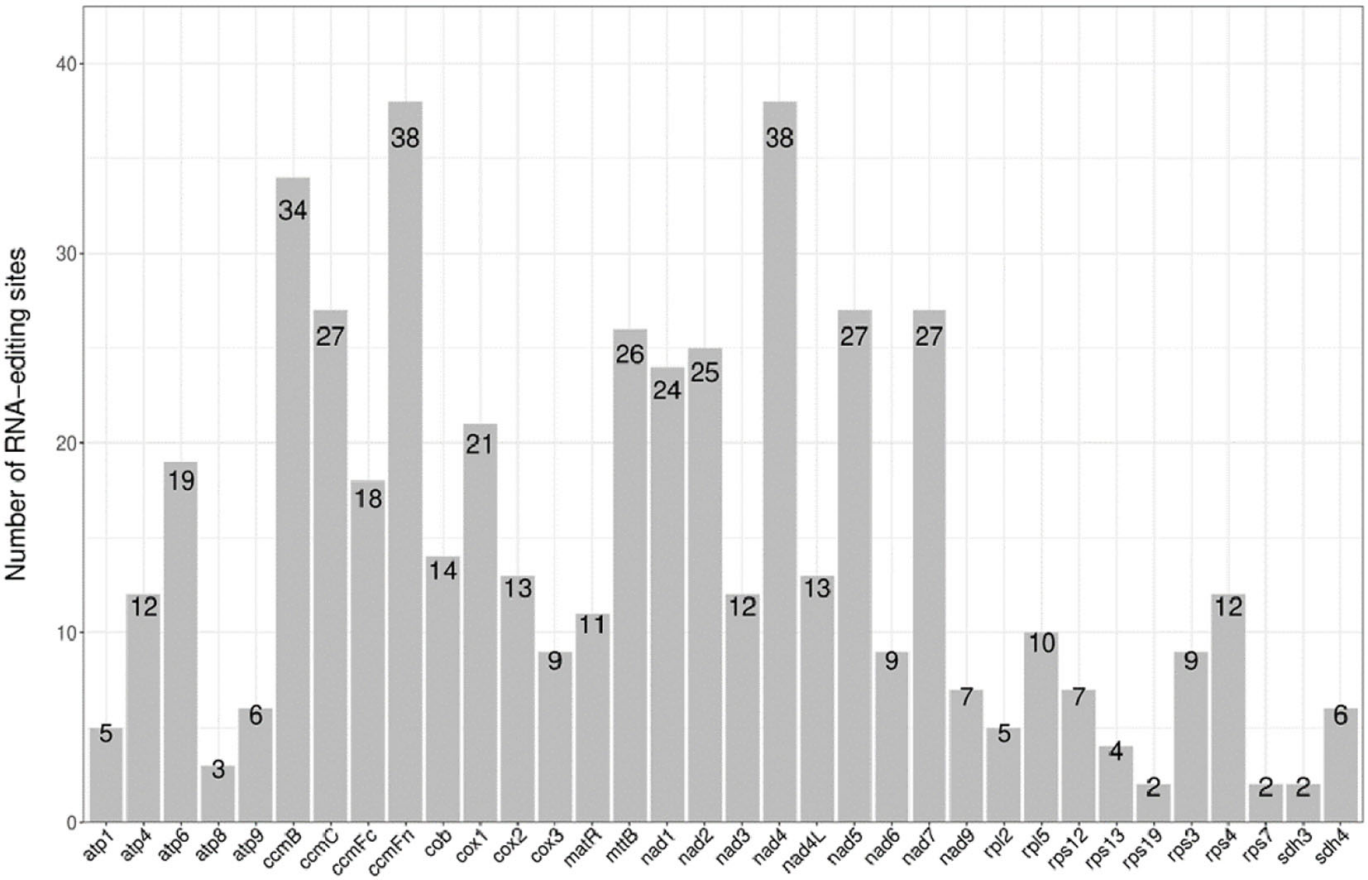
The distribution of RNA editing sites in mitogenome protein-coding genes of bottle gourd. The x-axis is the name of the gene. The y axis indicates the number of editing sites.

In this study, a total of 497 RNA editing sites were predicted across 34 protein-coding genes (PCGs) in the mitochondrial (mt) genome of *Lagenaria siceraria* (**Table 2**; **Figure 4**). Among these, the genes *rps19*, *rps7*, and *sdh3* had the fewest predicted editing sites, with only 2 each. In contrast, *ccmFn* and *nad4* contained the highest number of predicted editing sites, with 38 each. Following RNA editing, the hydrophobicity of 60.76% of the amino acids remained unchanged. However, 7.85% of hydrophobic amino acids were converted to hydrophilic, while 30.99% of hydrophilic amino acids became hydrophobic. All RNA editing events in the bottle gourd mt genome involved C-to-U conversions, with editing occurring at both the first and second positions of the triplet codon. This resulted in the conversion of proline (CCC) to phenylalanine (TTC or TTT). Notably, RNA editing in the coding genes *atp9* and *sdh4* led to premature termination of the coding process.

**Table 2:** Prediction of RNA editing sites.

Type	RNA-editing	Number	Percentage
Hydrophilic-hydrophilic	CAC (H) => TAC (Y)	8	
	CAT (H) => TAT (Y)	18	
	CGC (R) => TGC (C)	11	
	CGT (R) => TGT (C)	31	
	total	68	13.68%
Hydrophilic-hydrophobic	ACA (T) => ATA (I)	5	
	ACC (T) => ATC (I)	1	
	ACG (T) => ATG (M)	10	
	ACT (T) => ATT (I)	2	
	CGG (R) => TGG (W)	35	
	TCA (S) => TTA (L)	74	
	TCC (S) => TTC (F)	29	
	TCG (S) => TTG (L)	39	
	TCT (S) => TTT (F)	39	
	total	234	47.08%
Hydrophilic-stop	CGA (R) => TGA (X)	2	
	total	2	0.40%
Hydrophobic-hydrophilic	CCA (P) => TCA (S)	5	
	CCC (P) => TCC (S)	11	
	CCG (P) => TCG (S)	5	
	CCT (P) => TCT (S)	18	
	total	39	7.85%
Hydrophobic-hydrophobic	CCA (P) => CTA (L)	45	
	CCC (P) => CTC (L)	9	
	CCC (P) => TTC (F)	8	
	CCG (P) => CTG (L)	33	
	CCT (P) => CTT (L)	27	
	CCT (P) => TTT (F)	8	
	CTC (L) => TTC (F)	7	
	CTT (L) => TTT (F)	10	
	GCA (A) => GTA (V)	1	
	GCC (A) => GTC (V)	1	
	GCG (A) => GTG (V)	4	
	GCT (A) => GTT (V)	1	
	total	154	30.99%
	All	497	100%

### Analysis of repeats in the bottle gourd mitogenome

**Figure 5:**
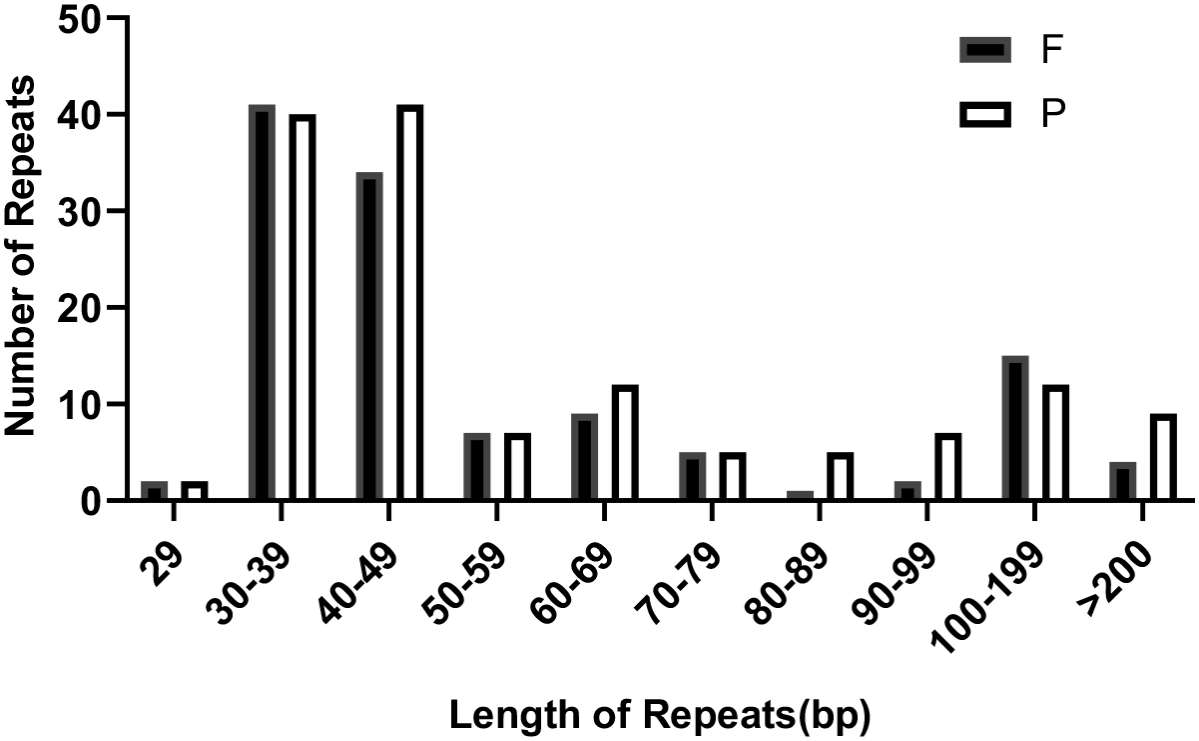
The length distribution of reverse and inverted repeats in the *L. siceraria* mt genome. F, Forward; P, Palindromic.

In the mitochondrial (mt) genome of bottle gourd, we identified a total of 260 interspersed repeats with lengths of 29 bp or greater. Among these, 123 were forward repeats, and 137 were palindrome repeats. The longest forward repeat sequence measured 2,349 bp, while the longest palindrome repeat sequence was 1,689 bp. As illustrated in **Figure 5**, forward repeats were most abundant in the 30–39 bp range, whereas palindrome repeats were most abundant in the 40–49 bp range.

A total of 100 simple sequence repeats (SSRs) were detected in the bottle gourd mitogenome. These included 32 (32%) mononucleotide repeats, 25 (25%) dinucleotide repeats, 9 (9%) trinucleotide repeats, 30 (30%) tetranucleotide repeats, and 4 (4%) pentanucleotide repeats (**Table 3**). Mononucleotide, tetranucleotide, and dinucleotide repeats were the most abundant types. Further analysis of SSR repeat units revealed that 90.63% of mononucleotide repeats consisted of A/T bases, and 72% of dinucleotide repeats were AT/TA. The high AT content of these SSRs contributes to the overall AT richness (54.97%) of the bottle gourd mitogenome. Additionally, as shown in **Table 4**, a total of 9 tandem repeats, ranging in length from 12 to 39 bp and with a match degree greater than 80%, were identified in the bottle gourd mitogenome.

**Table 3:** Distribution of SSRs in *L. siceraria* mt genome.

SSR type	Repeats	Numbers	Total
Monomer	A/T	29	32
	C/G	3	
Dimer	AC/GT	1	25
	AG/CT	13	
	AT/AT	11	
Trimer	AAG/CTT	5	9
	AAT/ATT	1	
	ACG/CGT	1	
	AGC/CTG	1	
	ATC/ATG	1	
Tetramer	AAAG/CTTT	9	30
	AAAT/ATTT	2	
	AAGC/CTTG	2	
	AAGG/CCTT	1	
	AAGT/ACTT	1	
	AATC/ATTG	1	
	AATG/ATTC	4	
	AATT/AATT	1	
	ACAT/ATGT	1	
	ACCG/CGGT	1	
	ACTG/AGTC	1	
	AGAT/ATCT	1	
	AGCG/CGCT	1	
	AGCT/AGCT	1	
	AGGG/CCCT	1	
	ATCC/ATGG	1	
	CCGG/CCGG	1	
Pentamer	AAAAG/CTTTT	1	4
	AAACT/AGTTT	2	
	ACTAG/AGTCT	1	

**Table 4:** Distribution of tandem repeats in *L. siceraria* mt genome.

NO.	Size	Copy	Repeat sequence	Percent matches	Start	End
1	18	2	TCTTCTCTTCTTGCTTAT	94	139805	139840
2	24	2.5	GACCGATAGGGAGAGGAGCAACTC	94	155235	155294
3	29	2.5	GAGGAGCGAAGCAGCTCGACCGATAGGGA	100	201828	201899
4	12	2.5	AAATGAATAATA	100	205993	206022
5	18	2.3	ACTATGAAACAGATCGCG	80	234448	234489
6	35	2.2	GAAGGAGCGAAGCAGCTTGACCGAGTTAGAGGG	90	238298	238370
7	26	2.2	GTAGTCTCTAGTTTGATATAGTAGTC	84	262583	262638
8	15	2	TACTAGGTCTTATGA	93	303623	303651
9	39	2	TTCACTCATGATCTGGCCTGGTCGACCCAATCATGATAT	97	336395	336473

### Ka/Ks analysis

**Figure 6:**
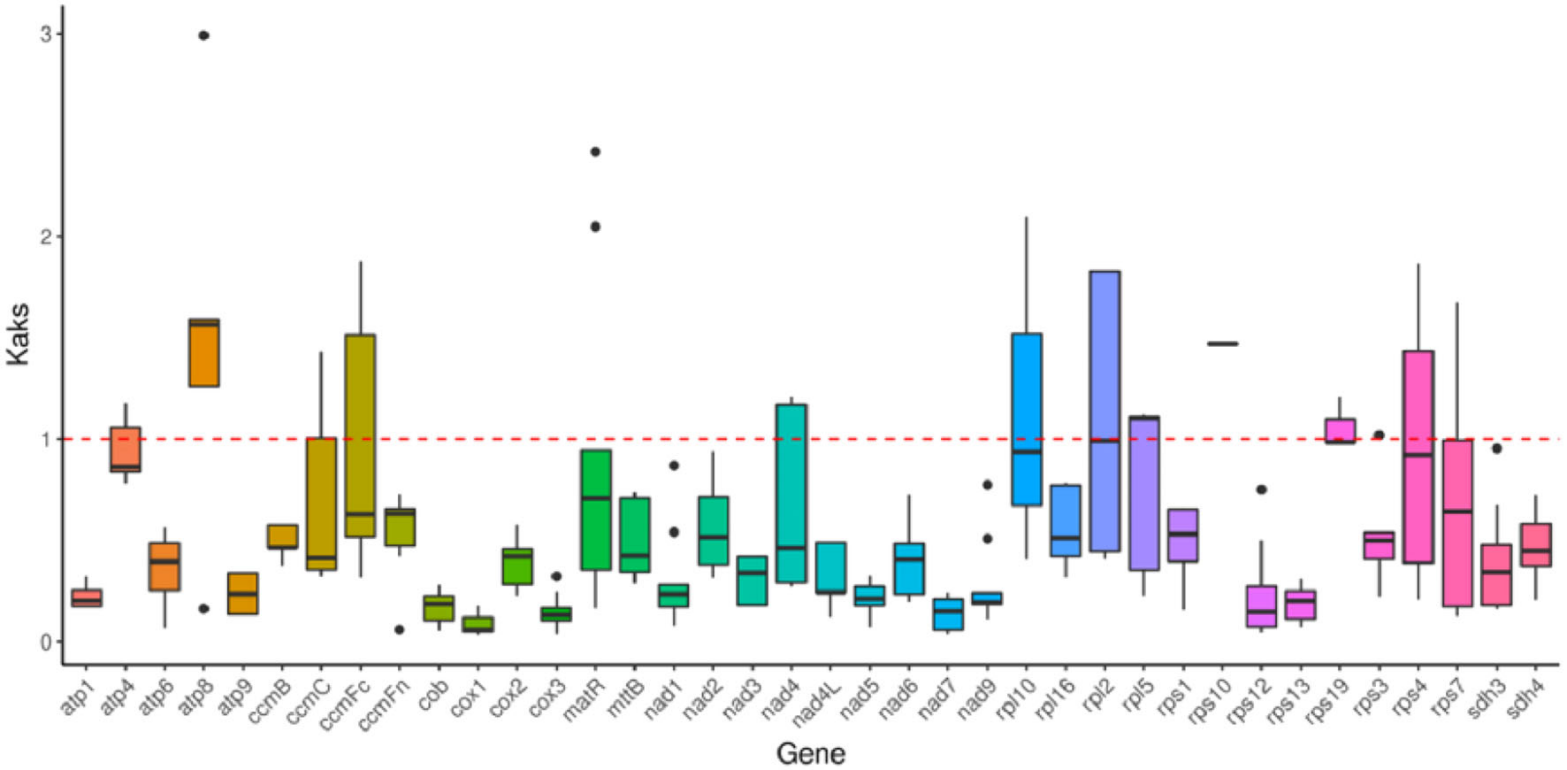
Ka/Ks ratios of 38 protein-coding genes in *L. siceraria*, *C. lanatus*, *C. sativus*, *L. acutangular* and *C. maxima.* Ka/Ks=1 means neutral selection. Ka/Ks > 1 indicates positive selection. Ka/Ks < suggests negative (purifying) selection.

In genetics, the nonsynonymous-to-synonymous substitution ratio (Ka/Ks) is a key metric for understanding the evolutionary dynamics of genes. The Ka/Ks ratio helps determine whether a protein-coding gene (PCG) is under selective pressure during evolution. Under neutral selection, Ka = Ks, resulting in a Ka/Ks ratio of 1. If Ka > Ks (Ka/Ks > 1), it indicates positive selection, whereas if Ks > Ka (Ka/Ks < 1), it suggests negative (purifying) selection. In this study, the Ka/Ks ratio was calculated for 38 PCGs shared among *L. siceraria*, *C. lanatus*, *C. sativus*, *L. acutangula*, and *C. maxima*. As shown in **Figure 6**, when comparing the mitochondrial (mt) genome of bottle gourd with that of *C. lanatus*, 16 PCGs exhibited Ka/Ks values < 1. In comparison to *C. sativus*, 24 PCGs had Ka/Ks values < 1, while 7 PCGs had Ka/Ks values > 1. Relative to *L. acutangula*, 15 PCGs showed Ka/Ks values < 1. When compared to *C. maxima*, 29 PCGs had Ka/Ks values < 1, and 3 PCGs had Ka/Ks values > 1. Notably, nearly all Ka/Ks ratios were less than 1.0, indicating that most PCGs were under stabilizing (purifying) selection during evolution. In contrast, two genes (*atp8* and *rps10*) had Ka/Ks ratios > 1.0, suggesting they underwent positive selection. Additionally, three genes (*atp4*, *rpl10*, *rpl2*, *rps19*, and *rps4*) had Ka/Ks ratios close to 1.

### Pi analysis

**Figure 7:**
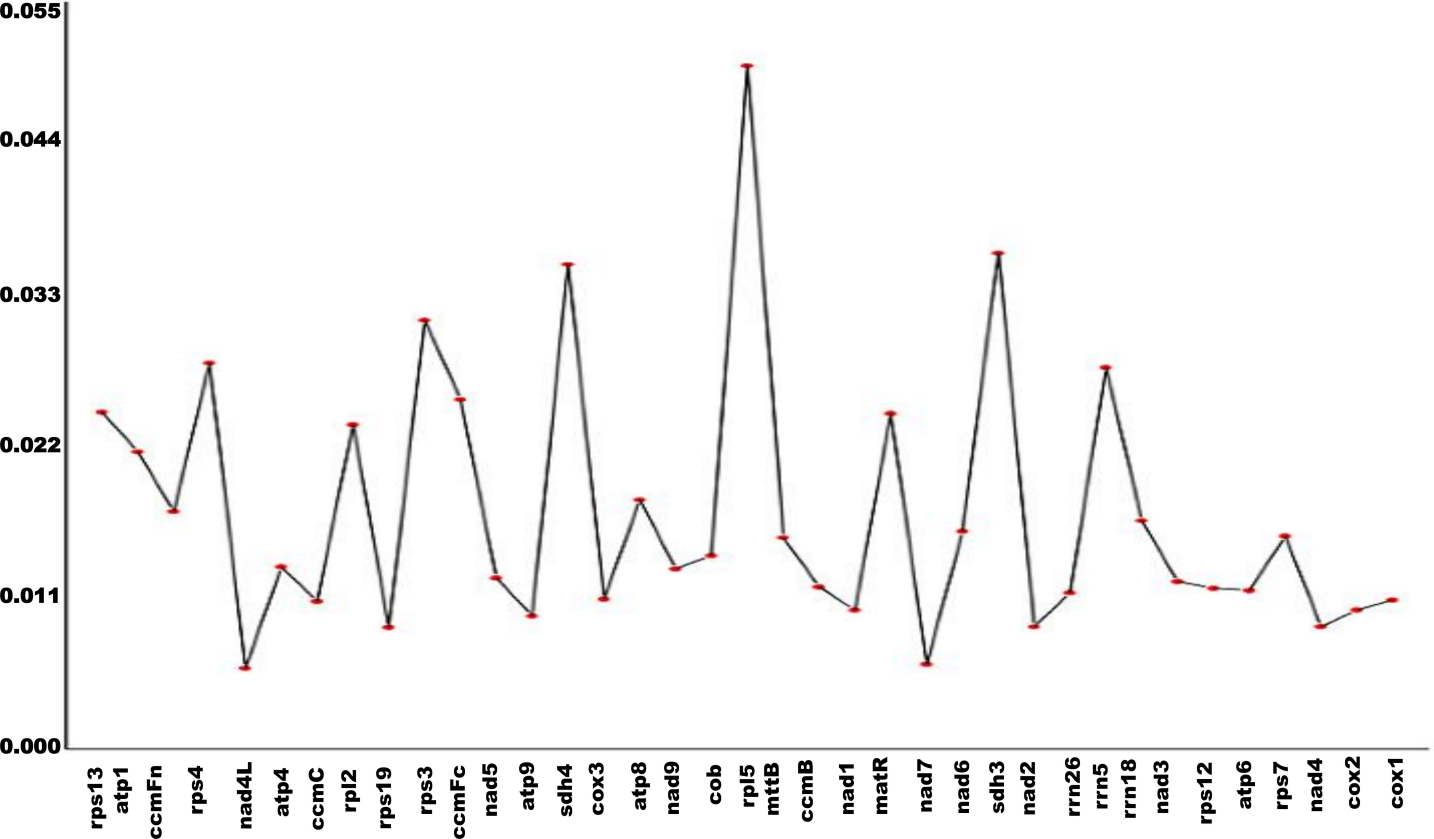
Nucleic acid diversity of genes in *L. siceraria.*The x-axis represents the gene name and the y-axis represents the pi value.

Nucleotide diversity (Pi) was calculated for 37 genes to assess sequence variation. A total of 1,338 polymorphic sites were identified (**Supplementary Table S1**). Among these, the maximum Pi value was 0.05028, corresponding to 65 polymorphic sites, while the minimum Pi value was 0.00594, associated with 4 polymorphic sites (**Figure 7**).

### Analysis of homologous fragments between mitochondria and chloroplasts

**Figure 8:**
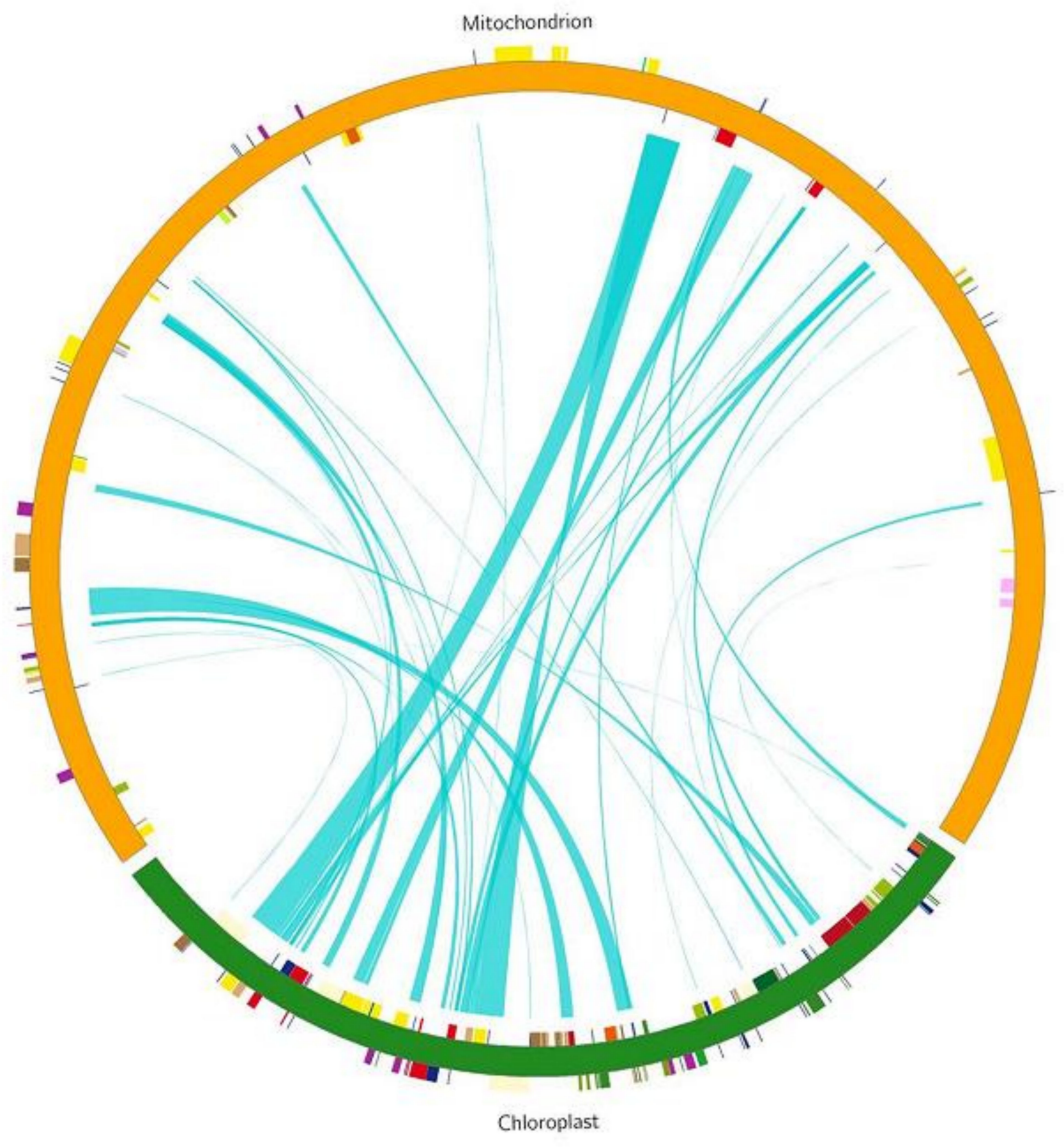
DNA and gene transfer between Chloroplast and Mitochondrial genomes in *L. siceraria*. The track shows complete genomes of cp and mt in green and orange respectively. The blue line segment in the circle connects the start and end points of the transferred gene fragments. The width of the blue line segment represents the size of the transferred fragment,.

We identified 45 homologous fragments between the mitochondrial (mt) and chloroplast (cp) genomes, with a total length of 40,579 bp, accounting for 11.35% of the mt genome (**Figure 8**, **Table 5**). These homologous fragments included 8 annotated genes, of which 6 were tRNA genes (*trnL-CAA*, *trnM-CAT*, *trnN-GTT*, *trnD-GUC*, *trnP-TGG*, and *trnV-GAC*) and 2 were ribosomal protein (SSU) genes (*rps7* and *rps12*).

**Table 5:** Comparison of a homologous fragment in the *L. siceraria* chl genome to that in the mt genome.

Number	Identity/%	Length(bp)	Mismatches	Gap openings	mt start	mt end	cp start	cp end	Gene
1	96.38	6195	122	37	143524	149663	226090	219943	rps7(partical:95.94%);ndhB;trnL-CAA;ycf2(partical:22.54%)
2	96.38	6195	122	37	94326	100465	219943	226090	ycf2(partical:22.54%);trnL-CAA;ndhB;rps7(partical:95.94%)
3	98.68	3109	32	5	68241	71342	49361	46255	petL(partical:54.17%);petG;trnW-CCA;trnP-TGG;psaJ;rpl33;rps18;rpl20
4	98.23	2426	28	7	80269	82679	49359	51784	rpoA;rps11;rpl36;rps8(partical:10.62%)
5	98.84	2240	20	2	123451	125684	242067	239828	ndhA(partical:55.54%);ndhH(partical:84.52%)
6	98.34	2170	30	3	111461	113624	108271	110440	trnN-GTT(partical:34.72%);NA;ndhF(partical:32.36%)
7	99.35	1530	10	0	130999	132528	109800	108271	ycf1(partical:0.64%);trnN-GTT(partical:34.72%)
8	96.63	1512	21	4	26899	28406	71338	72823	rpoB(partical:35.73%)
9	98.74	1274	10	2	101843	103115	272670	271402	ycf15;trnV-GAC
10	98.74	1274	10	2	140874	142146	271402	272670	trnV-GAC;ycf15
11	97.52	1009	16	1	147	1155	238838	239837	psbA(partical:83.90%)
12	99.89	922	1	0	34430	35351	147610	148531	psbD(partical:57.91%)
13	99.31	871	6	0	136830	137700	44869	43999	rrn23S(partical:3.02%);trnA-TGC(partical:72.46%)
14	99.31	871	6	0	106289	107159	43999	44869	trnA-TGC(partical:72.46%);rrn23S(partical:3.02%)
15	97.9	808	13	1	122456	123259	242869	242062	ndhA(partical:35.87%)
16	97.88	708	11	1	142700	143403	226843	226136	rps12(trans_splicing)(partical:79.28%)
17	97.88	708	11	1	100586	101289	226136	226843	rps12(trans_splicing)(partical:88.22%)
18	100	411	0	0	103687	104097	118894	118484	rrn16S(partical:27.57%)
19	100	411	0	0	139892	140302	118484	118894	rrn16S(partical:27.57%)
20	97.63	421	6	1	26250	26666	274391	273971	rpoB(partical:12.98%)
21	99.11	337	2	1	25882	26217	274728	274392	rpoB(partical:10.46%)
22	99.21	253	0	1	105095	105345	108288	108036	trnI-GAT(partical:28.27%)
23	99.21	253	0	1	138644	138894	108036	108288	trnI-GAT(partical:28.27%)
24	77.46	732	93	38	31442	32145	326735	326048	trnD-GTC
25	73.93	886	182	38	103514	104377	255533	254675	rrn16S(partical:57.95%)
26	73.93	886	182	38	139612	140475	254675	255533	rrn16S(partical:57.95%)
27	96.47	170	4	2	142364	142531	266823	266654	ORF
28	96.47	170	4	2	101458	101625	266654	266823	ORF
29	89.6	173	16	2	68887	69057	238575	238403	trnP-TGG
30	97.62	126	3	0	101605	101730	272908	272783	ORF
31	97.62	126	3	0	142259	142384	272783	272908	ORF
32	94.74	133	7	0	142140	142272	272789	272657	ORF
33	94.74	133	7	0	101717	101849	272657	272789	ORF
34	89.87	148	15	0	44613	44760	119709	119562	ycf3(partical:6.95%)
35	99.03	103	1	0	35764	35866	278975	279077	psbD(partical:3.30%);psbC(partical:7.24%)
36	83.93	168	19	8	68643	68805	238803	238639	trnW-CCA
37	95.6	91	2	2	35	124	34051	33962	trnH-GTG
38	94.12	85	4	1	111409	111492	91551	91467	trnN-GTT
39	94.12	85	4	1	132497	132580	91467	91551	trnN-GTT
40	94.94	79	4	0	54563	54641	288026	288104	trnM-CAT
41	97.06	68	2	0	25879	25946	250462	250529	rpoB(partical:2.12%)
42	100	48	0	0	102992	103039	185599	185552	trnV-GAC(partical:52.78%)
43	100	48	0	0	140950	140997	185552	185599	trnV-GAC(partical:52.78%)
44	97.44	39	1	0	11746	11784	338148	338186	atpA(partical:2.56%)
45	81.61	87	4	6	88858	88932	40751	40665	trnI-CAT
46	81.61	87	4	6	155057	155131	40665	40751	trnI-CAT

### Phylogenetic analysis and gene arrangement analysis

**Figure 9:**
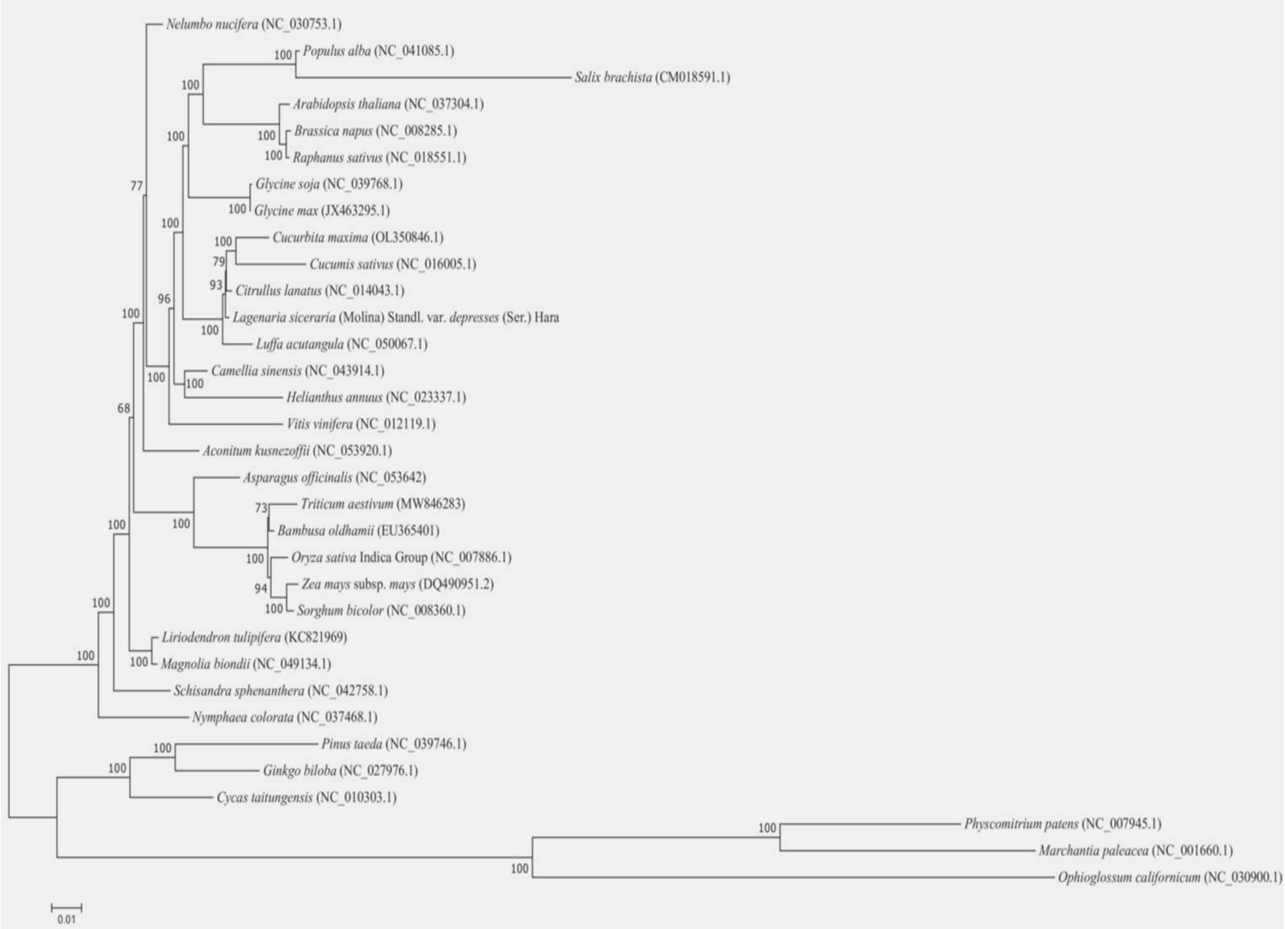
The phylogenetic relationships of *L. siceraria* with other 32 plant species. *C. maxima*, *C. sativus*, *C. lanatus*, and *L. acutangula* were closely clustered with bottle gourd.

Phylogenetic trees were constructed using the maximum likelihood method to explore the evolutionary relationships between the bottle gourd mt genome and the published mt genomes of 32 plant species. The selected species and their details are listed in **Table 6**. The results revealed that *C. maxima*, *C. sativus*, *C. lanatus*, and *L. acutangula* were closely clustered with bottle gourd (**Figure 9**).

**Table 6:** NCBI accession numbers of mt genomes used in this study.

Species	Family	Category	Accession number	Size
*Nelumbo nucifera*	Nelumbonaceae	*Nelumbo*	NC_030753.1	524,797 bp
*Populus alba*	Saliceae	*Populus*	NC_041085.1	838,420 bp
*Salix brachista*	Saliceae	*Salix*	CM018591.1	608,983 bp
*Arabidopsis thaliana*	Cruciferae	*Arabidopsis*	NC_037304.1	367,808 bp
*Brassica napus*	Cruciferae	*Brassica L.*	NC_008285.1	221,853 bp
*Raphanus sativus*	Cruciferae	*Raphanus L.*	NC_018551.1	258,426 bp
*Glycine soja*	Fabaceae	*Glycine*	NC_039768.1	402,545 bp
*Glycine max*	Fabaceae	*Glycine*	JX463295.1	402,558 bp
*Cucurbita maxima*	Cucurbiteae	*Cucurbita*	OL350846.1	640,814 bp
*Cucumis sativus*	Cucurbiteae	*Cucumis*	NC_016005.1	1,555,935 bp
*Citrullus lanatus*	Cucurbiteae	*Citrullus*	NC_014043.1	379,236 bp
*Luffa acutangula*	Cucurbiteae	*Luffa*	NC_050067.1	460,333 bp
*Camellia sinensis*	Theaceae	*Camellia*	NC_043914.1	707,441 bp
*Helianthus annuus*	Heliantheae	*Helianthus*	NC_023337.1	300,945 bp
*Vitis vinifera*	Viteae	*Vitis*	NC_012119.1	773,279 bp
*Aconitum kusnezoffii*	Delphinieae	*Aconitum*	NC_053920.1	440,720 bp
*Asparagus officinalis*	Asparagoideae	*Asparagus*	NC_053642	492,062 bp
*Triticum aestivum*	Triticinae	*Triticum*	MW846283	452,526 bp
*Bambusa oldhamii*	Bambusinae	*Bambusa*	EU365401	509,941 bp
*Oryza sativa Indica Group*	Oryzinae	*Oryza*	NC_007886.1	491,515 bp
*Zea mays subsp. mays*	Tripsacinae	*Zea*	DQ490951.2	557,162 bp
*Sorghum bicolor*	Sorghinae	*Sorghum*	NC_008360.1	468,628 bp
*Liriodendron tulipifera*	Magnoliaceae	*Liriodendron*	KC821969	553,721 bp
*Magnolia biondii*	Magnoliaceae	*Magnolia*	NC_049134.1	967,100 bp
*Schisandra* sp*henanthera*	Schisandraceae	*Schisandra*	NC_042758.1	1,101,768 bp
*Nymphaea colorata*	Nymphaeaceae	*Nymphaea*	NC_037468.1	617,195 bp
*Pinus taeda*	Pinaceae	*Pinus*	NC_039746.1	1,191,054 bp
*Ginkgo biloba*	Ginkgoaceae	*Ginkgo*	NC_027976.1	346,544 bp
*Cycas taitungensis*	Cycadaceae	*Cycas*	NC_010303.1	414,903 bp
*Physcomitrium patens*	Funariaceae	*Physcomitrium*	NC_007945.1	105,340 bp
*Marchantia paleacea*	Marchantiaceae	*Marchantia*	NC_001660.1	186,609 bp
*Ophioglossum californicum*	Ophioglossoideae	*Ophioglossum*	NC_030900.1	372,339 bp

Based on the phylogenetic tree, the 32 plant species were grouped into three major clusters: angiosperms, gymnosperms, and spore plants. The clustering pattern in the phylogenetic tree aligns with the traditional taxonomic relationships at the family and genus levels, demonstrating the reliability of mt genome-based phylogenetic analysis.

**Figure 10:**
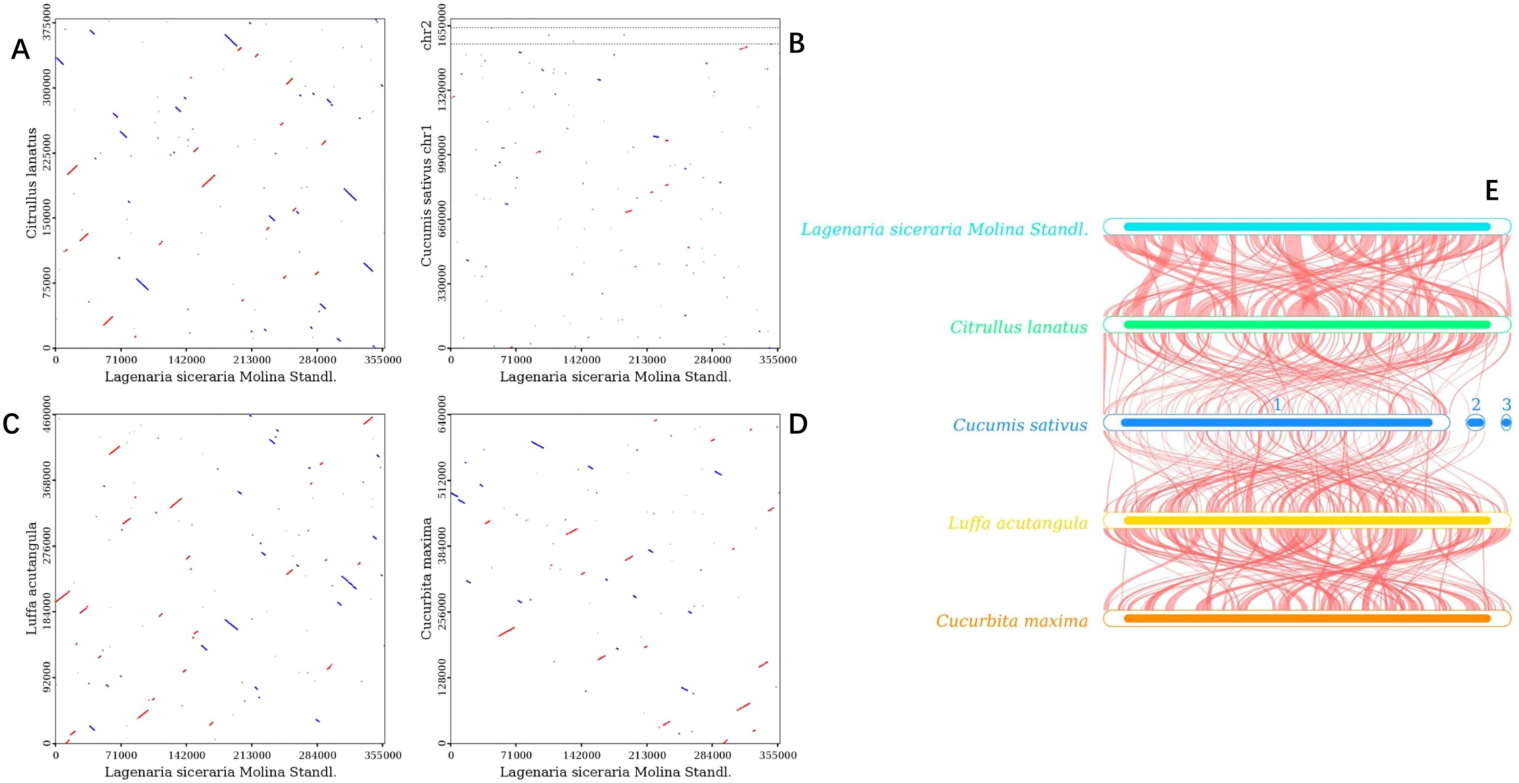
Collinearity analysis of the mitogenomes of *L. siceraria*, *C. sativus*, *C. maxima*, *C. lanatus* and *L. acutangular*. **(A–D)** are dot plots of *C. lanatus*, *C. sativus*, *L. acutangular*, and *C. maxima* with *L. siceraria*, respectively. **(E)** *L. siceraria* mitogenome synteny. The box in each row represents a genome, and the connecting line in the middle represents homology regions.

Dot plot analysis revealed only sporadic collinear regions between *C. sativus* and *L. siceraria*, indicating poor collinearity (**Figure 10B**). In contrast, *C. maxima*, *C. lanatus*, and *L. acutangula* exhibited better collinearity with *L. siceraria* (**Figures 10A, C, D**). These findings were further supported by BLASTn collinearity analysis (**Figure 10E**).

## Discussion

The size of mitochondrial genomes varies significantly among different species. Previous studies have shown that angiosperms possess larger mitochondrial genomes than animals (Best et al., 2020; Christensen, 2013). To date, *Silene conica* (11.3 Mb) has the largest known mitochondrial genome in plants. Among cucurbit crops, the mitochondrial genome size ranges from 379 kb to 2,936 kb, with *C. melo* having the largest genome (Alverson et al., 2010). The mitochondrial genome of bottle gourd is 357,496 bp, smaller than that of watermelon (379,236 bp), making it the smallest mitochondrial genome among cucurbit crops. Although plant mitochondrial genomes are large, they typically contain only 50–60 coding genes, as the coding regions account for only 7–17% of the total genome, with the remainder consisting of intergenic regions. In bottle gourd, the coding region constitutes 8.48% of the mitochondrial genome, with 34 protein-coding genes. Watermelon, in contrast, has 37 protein-coding genes, similar to bottle gourd (Alverson et al., 2010). GC content is another important indicator for species evaluation (Liu et al., 2023). The GC content of cucurbit crops generally ranges from 44.1% to 44.6%, with cucumber mitochondrial genomes having a GC content of 44.2–44.6%, *Cucumis hystrix* at 44.5%, and *Cucumis melo* at 44.1%. However, bottle gourd has a higher GC content of 45.03%, the highest among known cucurbit crops.

Previous comparative analyses of mitochondrial genome sequences in cucurbit crops have revealed the presence of unique conserved sequences. A comparative analysis of mitochondrial genome composition between bottle gourd and other cucurbit species showed that bottle gourd possesses the *rps19* gene, which is present in most species. However, the *rpl10* gene, found in *C. melo*, *C. hystrix*, and *C. sativus* (Xia et al., 2022) is absent in the mitochondrial genome of bottle gourd. The mitochondrial rpl10 gene has become a pseudogene in some plants and has been entirely lost from the mitochondrial genome in others. The lost mitochondrial rpl10 gene has been replaced by an extra copy of the nuclear gene that normally encodes chloroplast rpl10 protein (Kubo and Arimura, 2009). The loss of rpl10 in the mitochondria of bottle gourd and its existence in the others indicate that the evolution of rpl10 within cucurbit crops has taken some unexpected and interesting turns. Additionally, the number of tRNA genes varies significantly among species, with 40 in *C. melo*, 13 in *C. pepo*, and 24 in bottle gourd. This suggests that tRNA genes have undergone substantial changes during the evolution of cucurbit crops. The presence of extra tRNA and *rps* genes in bottle gourd, which originated from chloroplast horizontal gene transfer, distinguishes it from other cucurbit species. This implies that sequence transfer between plastid genomes is a frequent occurrence during the evolution of flowering plants (Notsu et al., 2002; Xia et al., 2022). These transfer events contribute to the acquisition of functional tRNA genes and help explain the genetic variation observed in mitochondrial genomes across higher plants (Alverson et al., 2011; Xia et al., 2022).

Codon usage analysis indicates that, as in most other plants, Leu, Ser, and Arg are the most common amino acids in bottle gourd, while Met and Trp are much less frequent (**Figure 2**) (Ma et al., 2022). The preference for codons ending in A/T in the bottle gourd mitochondrial genome aligns with the codon usage patterns of most dicotyledons, in contrast to monocotyledons, which favor codons ending in G/C (Mazumdar et al., 2017). RNA editing, another critical factor influencing gene expression in plant mitochondrial genomes, plays a significant role in plant evolution (Edera et al., 2018). In cucurbit crops, RNA editing typically occurs at one of the first two positions of the codon, with the number of editing sites ranging from 444 to 501. In bottle gourd, 497 RNA editing sites were identified, a number similar to that found in *C. hystrix* (501) (Xia et al., 2022). RNA editing can take various forms, such as C-to-U, U-to-C, and A-to-I conversions (Small et al., 2019). However, in bottle gourd, all RNA editing events involve C-to-U conversions (**Table 2**), consistent with the pattern observed in *C. hystrix*. High-frequency RNA editing serves as a critical strategy for mitochondria to cope with genomic reduction, environmental stress, and complex regulatory demands, reflecting the profound evolutionary significance of post-transcriptional regulation in bottle gourd. This mechanism balances the stability and flexibility of genetic information, holding key value for understanding cellular metabolism and evolution.

Repetitive sequences in plant mitochondria play a crucial role in determining genome size, structure, and recombination (Cole et al., 2018). In bottle gourd, we identified multiple interspersed repeats, simple sequence repeats (SSRs), and tandem repeats. The total length of repetitive sequences in the bottle gourd mitochondrial genome is 22,294 bp, accounting for 6.24% of the genome. Compared to other cucurbit crops, bottle gourd has the fewest repetitive sequences, which may explain why its mitochondrial genome is the smallest among cucurbit species.

The results of Ka/Ks analysis of the mt genomes of *L. siceraria*, *C. lanatus*, *C. sativus*, *L. acutangula*, and *C. maxima* that most of the genes were negatively selected during the evolution process, indicating that the protein-coding genes of the bottle gourd mt genome are relatively well-conserved. However, the positive selection on *atp8* and *rps10* may enhance energy metabolism efficiency and translational capacity, thereby improving adaptability to growth or environmental stress in bottle gourd. This hypothesis requires further validation through combined experimental and evolutionary analyses, offering new insights into the domestication mechanisms of mitochondrial genes in crops.

DNA transfer between organelles, as well as between nuclear genomes and species, is a common phenomenon in plants. However, the extent of such transfers varies significantly among species (Timmis et al., 2004). Reported cases range from 50 kb in *A. thaliana* to 1.1 Mb in *Oryza sativa subsp. Japonica*. In this study, we identified 40,579 bp of DNA transferred from the chloroplast (cp) genome to the mitochondrial (mt) genome, accounting for 11.35% of the mt genome. This proportion is higher than that observed in other crops, such as *Bupleurum chinense* DC (2.56%), *Acer truncatum* (2.36%), and *Suaeda glauca* (5.18%) (Qiao et al., 2022).

The mitochondrial genome serves as a valuable source of genetic information for phylogenetic research (Xia et al., 2022). In this study, *C. maxima*, *C. sativus*, *C. lanatus*, *L. acutangular*, *and L. siceraria* were grouped together in the *Cucurbiteae* family. The topology of the mitochondrial DNA-based phylogenetic tree aligns with the Angiosperm Phylogeny Group classification. The clustering of these 32 species on the evolutionary tree is consistent with their traditional taxonomic relationships, demonstrating the congruence between traditional and molecular taxonomy. While cucumber and bottle gourd fruits are typically used as vegetables, and watermelon fruits are consumed as fruits, evolutionary analysis reveals that bottle gourd is more closely related to watermelon than to cucumber. This is further supported by similarities in genome size, composition, and the number of repetitive sequences between bottle gourd and watermelon.

## Conclusion

We present the first complete mitochondrial genome assembly and annotation of a cucurbit crop, bottle gourd. The mitochondrial genome of gourd is also the smallest among cucurbitaceae crops so far.Comparative analysis of gene structure, codon usage, repeat regions, and RNA editing sites in the bottle gourd mitochondrial genome were analyzed, contributing to our understanding of bottle gourd. Repeat sequences, RNA editing edits, and the horizontal gene transfer events in the bottle gourd mitochondrial genome were analyzed, contributing to our understanding of bottle gourd.We found that bottle gourd is closely related to watermelon in size, but *L. acutangula* exhibits the highest collinearity with *L. siceraria* according to gene arrangement analysis. Further resolution of mitochondrial genomic information could contribute to our knowledge of the unique mitochondrial revolution of bottle gourd. The well-conserved protein-coding genes in mitochondrial genome of the bottle gourd could potentially serve as molecular markers in phylogenetic studies. This study provides extensive information about the mitochondrial genome for *L. siceraria*, facilitating the deciphering of evolutionary and genetic relationships within the cucurbit crops.

## Data Availability

The datasets presented in this study can be found in online repositories. The names of the repository/repositories and accession number(s) can be found in the article/**Supplementary Material**.
